# Immune Checkpoint Inhibition in Head and Neck Cancer

**DOI:** 10.3389/fonc.2018.00310

**Published:** 2018-08-29

**Authors:** Martin David Forster, Michael-John Devlin

**Affiliations:** Department of Oncology, UCL Cancer Institute, London, United Kingdom

**Keywords:** immune checkpoint inhibitors, head and neck cancer, head and neck squamous cell carcinoma, cancer immunology, cancer immunotherapy

## Abstract

Head and Neck Squamous Cell Carcinoma (HNSCC) is the 6th most common cancer globally and commonly presents with locally advanced disease, which has a recurrence rate of around 50% despite aggressive multi-modality treatment involving surgery, radiotherapy and chemotherapy or EGFR inhibition where appropriate. As understanding of the underlying cancer biology and the complex interactions within the tumor microenvironment improves, there is gathering interest in and evidence for the role of immunomodulating agents in the management of HNSCC. Immune checkpoint inhibitors, which aim to hinder the inhibitory interaction between programmed cell death protein 1 (PD-1) and its ligand PD-L1, have demonstrated durable improvements in patient outcomes in advanced / metastatic HNSCC, with both pembrolizumab and nivolumab being granted FDA approval in 2016. There are numerous ongoing clinical trials exploring the role of checkpoint inhibitors both as single agents and in combination, administered with established treatment modalities such as chemotherapy and radiotherapy, as well as alongside other novel immune modulators. These trials are not limited to advanced / metastatic HNSCC, but also to the neo-adjuvant or adjuvant settings. As studies complete and more results become available, the role immunotherapy agents will have within the treatment strategies for HNSCC may change, with increasing biomarker selection resulting in personalized therapy aiming to further improve patient outcomes.

Head and neck cancer encompasses malignancies that arise in the paranasal sinuses, nasal cavity, oral cavity, pharynx and larynx. In Europe, there are around 139,000 new cases of head and neck cancer per year, 90% of which are squamous cell carcinoma (HNSCC) (1). While both smoking and alcohol consumption have long been established as risk factors for the development of HNSCC, human papillomavirus (HPV) has emerged as a driver for a significant proportion of oropharyngeal squamous cell carcinomas and increasingly is being recognized as its own distinct clinical entity with a more favorable prognosis than non-HPV associated HNSCC (2, 3).

The 2016 FDA approval of the Programmed death-1 (PD-1) monoclonal antibodies pembrolizumab and nivolumab for the treatment of HNSCC heralded the dawn of a new era of treatment for a patient population that historically has a 50% recurrence rate despite aggressive multi-modality treatment involving surgery, radiotherapy, chemotherapy and, where appropriate, EGFR inhibition (4).

Intact immune surveillance is critical to control carcinogenesis and in order to propagate locally and metastasise cancer cells must develop mechanisms that allow them to evade elimination by the host immune system. Immunotherapy works on the premise that the host immune system can be activated to overcome these acquired mechanisms, allowing the recognition of cancer as non-self and eliminate it.

In order for this to happen, T-lymphocytes must be able to infiltrate the tumor and mount appropriate responses (5) and higher numbers of CD3+, CD8+ and FOXP3+ tumor infiltrating lymphocytes (TILs) are associated with a favorable outcome in in several malignancies, including HNSCC (6). In particular, the presence of CD8+ “effector” T-cells and the ratio between CD8+ and FOXP3+ regulatory T-cells (Tregs) correlates with improved prognosis. Tregs are known to actively suppress immune responses (7) and as such their presence within the tumor microenvironment may assist immune evasion in head and neck cancers. However, further understanding is required as although their presence is linked to a decreased survival in several tumor types, a positive correlation has been reported in other cancers such epithelial ovarian cancer, colorectal cancer and lymphoma (8–10).

Some cancers evade T-cell directed immune effects by developing ways of excluding the T-cells from the tumor microenvironment. However, HNSCC has been found to be one of the most immune-infiltrated cancer types (11) suggesting other mechanisms are involved and T-cell homing, infiltration and activity are under a number of influences generated by the tumor. In HNSCC several suppressive mechanisms have been identified that include:

1. Deficiencies or alterations of tumor human leukocyte antigen (HLA) class I molecules expression (12, 13) along with overexpression of antigens causing T-cell tolerance (14)
2. Increases in immunosuppressive cytokines such Il-10, (15) IL-6 (16), and TGF-β (17)
3. Aberrant activation of the transcription factors Signal Transducers and Activators of Transcription 3 (STAT3) (18) and NF-kB, (17) which are notably linked to IL-6 and TGF-β signaling respectively.

To avoid autoimmunity a series of checkpoints exist on the surface of immune cells, with the activation of a T-cell response being a careful balance of co-inhibitory and co-stimulatory molecules and their ligands (19) (Figure 1). In HNSCC immunomodulatory agents exist that block interaction between co-inhibitory receptors such as Cytotoxic T-Lymphocyte-associated antigen 4 (CTLA-4), Programmed death-1 (PD-1) or Lymphocyte activation gene-3 (LAG-3) and their ligands. Conversely, biologics that mimic ligand activated signaling in co-stimulatory molecules such as Glucocorticoid-induced tumor necrosis factor receptor (GITR) have also been evaluated. Both these approaches aim to achieve the same outcome; an enhanced activation of an immune response to tumor cells (Figure 2).

**Figure 1:**
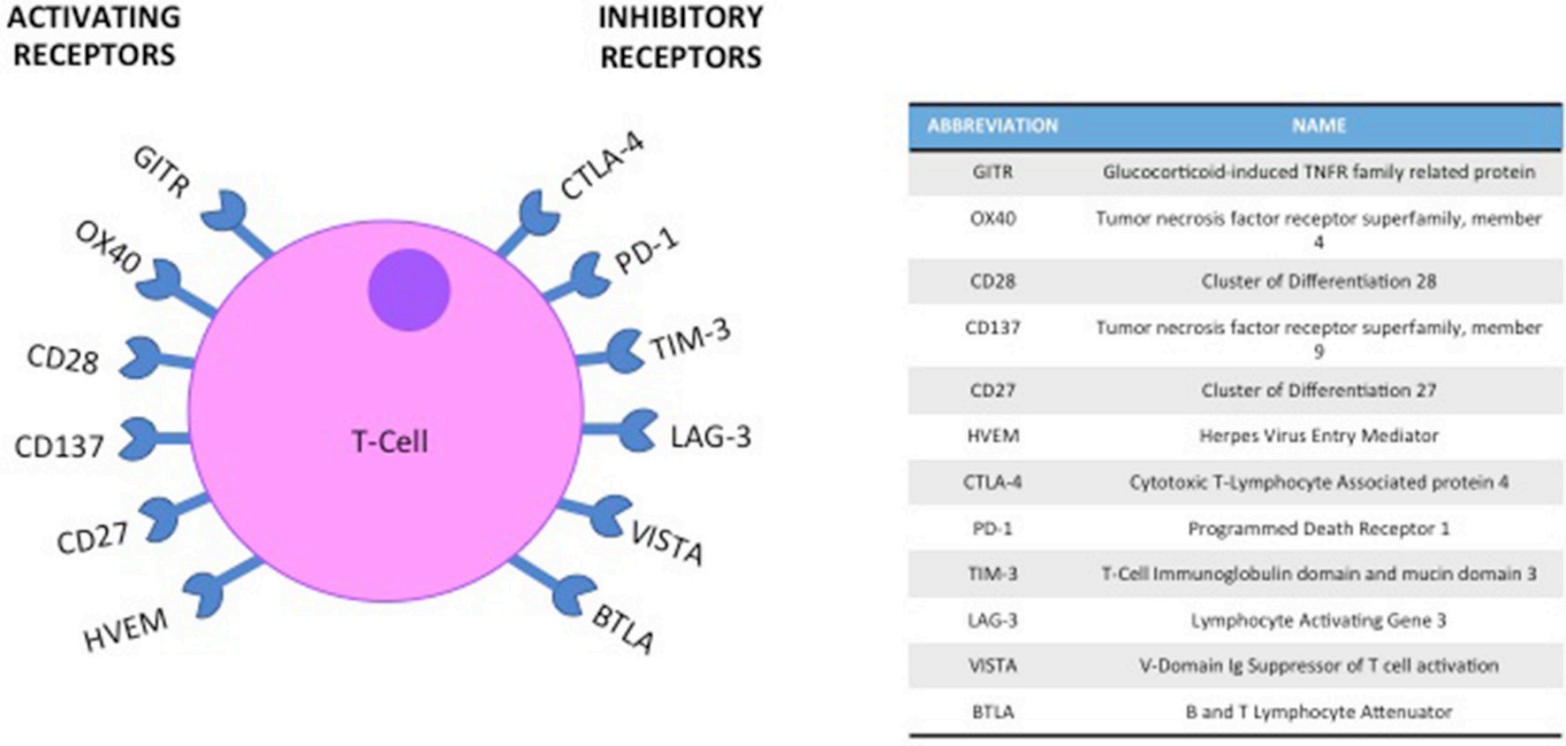
Co-inhibitory and co-stimulatory checkpoints are a key element of T-cell immune regulation.

**Figure 2:**
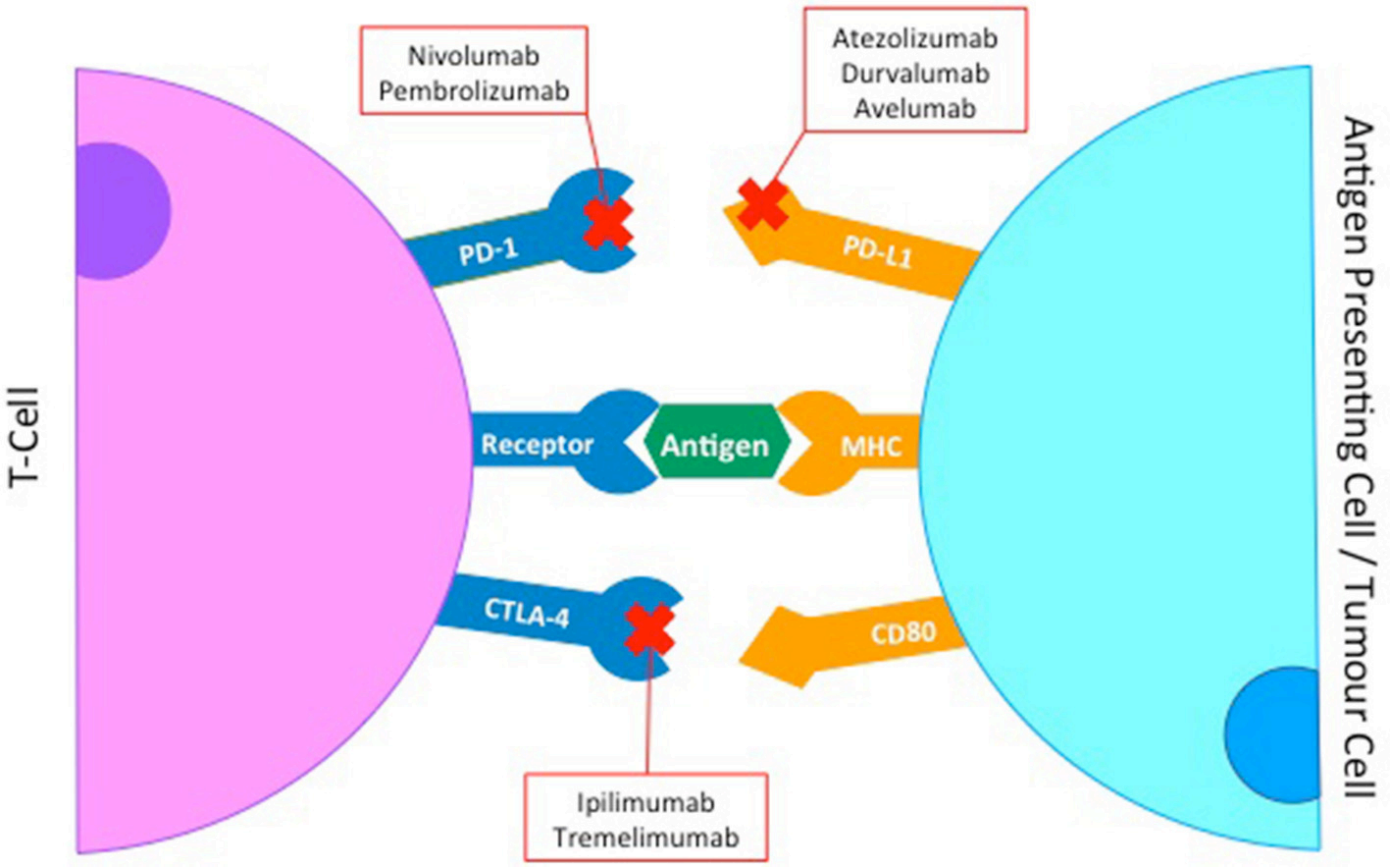
Monoclonal antibodies to key co-inhibitory immune checkpoints.

## PD-1/PD-L1 axis

PD-1 is a member of the CD28 receptor family which is expressed on activated T- and B-cells, monocytes and a subset of thymocytes. The expression of PD-1 on activated T cells enhances the suppressive function of Tregs and this effect is mediated by the interaction with its ligands, PD-L1 and PD-L2, which are expressed on antigen presenting cells, endothelial and epithelial cells as well as on activated lymphocytes. The interaction between PD-1 and PD-L1 negatively regulates immune responses by decreasing cytokine production and inducing T lymphocyte anergy and apoptosis. Upregulation of PD-L1 can occur in tumor cells and allows cancer cells to escape from host immune systems by functionally inactivating T-cell immune surveillance. The inhibition of this interaction can enhance T-cell response and mediate clinical anti-tumor activity (20, 21). Expression of PD-L1 is often high in HNSCC tumors, with positivity being quoted between 46 and 100% across several studies, this wide range likely owing to differences in staining technique, sample preservation and possibly sampling error (22).

Nivolumab is an IgG4 PD-1 monoclonal antibody designed to block co-inhibitory signaling through the PD-1/PD-L1 axis. Following early phase studies demonstrating promising data, a phase III trial, CheckMate 141, compared nivolumab with the physician's choice of second line single agents (docetaxel, methotrexate or cetuximab) in a 2:1 randomisation in patients with platinum resistant recurrent / metastatic HNSCC. It recruited 361 patients and response rates (RR) were low in all arms, but higher in the nivolumab cohort (13.3%) with 6 complete responses (CR) and 26 partial responses (PR). In the chemotherapy arm, RR was 5.8%, including 1 CR and 6 PR. The median overall survival (OS) was 7.5 months with nivolumab, vs. 5.1 months in the control arm, and patients who received nivolumab had a statistically significant 30% lower risk of death (HR 0.70; 95% CI 0.51 to 0.96). There was also an improvement in the estimated progression free survival (PFS) at 6 months; 19.7% with nivolumab and 9.9% for those who received physician's choice. This increased benefit at later time-points gives promise to durable benefit for responding patients, as seen with nivolumab in other tumor types where clinical data are more mature (23). In addition to the higher efficacy seen, treatment with nivolumab was also more tolerable than the standard therapies, with a reduction in grade 3 or 4 adverse events; 13.1 vs. 35.1% and a relative improvement in patient reported outcomes (PROMS) and other Quality of Life parameters (24). Outcomes of patients randomized to nivolumab who were Treated Beyond Progression (TBP) were presented at the European Society of Medical Oncology 2017 Annual Congress (ESMO 2017). Of those patients who had progressed, 62 patients (42%) received at least one further dose of nivolumab and of these 15 (24%) had a subsequent reduction in target lesion size with 3 patients achieving >30% reduction, with no increase in grade 3 or 4 toxicities (25). These findings of unusual patterns of response to immune checkpoint inhibitors have also been described in other patient groups (26, 27) and offers reassurance that continuing therapy beyond progression is safe and may have a role to play in patients maintaining clinical benefit.

Pembrolizumab, another IgG4 PD-1 antibody, has also been examined as a treatment for patients with recurrent or metastatic HNSCC. KEYNOTE-012 was a phase Ib study which included an expansion cohort of 132 HNSCC patients. A response rate of 18% at 9 months was reported (including 4 CR and 20 PR), with a median OS of 8 months and a 6-month PFS of 23%. Treatment was well tolerated with grade 3 or 4 toxicity reported in 9% of patients (28) These data led to the accelerated approval of pembrolizumab by the FDA. Outcomes from the confirmatory phase III trial comparing pembrolizumab with standard therapies (KEYNOTE-040) in patients with platinum-resistant relapsed / metastatic head and neck squamous cell cancers were also presented at ESMO 2017 (29), although the final published manuscript is awaited. This study was similar in design to the CheckMate 141 study, although with a complex hierarchical statistical analysis plan. It demonstrated a median OS of 8.4 months with pembrolizumab compared to 7.1 months in the control arm, with a hazard ratio of 0.81 (95% confidence interval 0.66–0.99), which did not reach statistical significance (*p* = 0.020) within the multiplicity statistical model. The overall survival data may have been confounded by some patients (12%) within the control arm going on to receive immune checkpoint inhibitors post progression (cross-over effect). The evaluation of other inhibitors of the PD-1/PD-L1 axis in HNSCC, such as durvalumab, atezolizumab and avelumab, are currently ongoing.

## GITR

GITR is a member of the Tumor Necrosis Factor superfamily, expressed on the surface of CD25+ CD4+ Tregs. GITR activation by its ligand (GITRL) reduces Treg recruitment and abrogates their suppressive function (30, 31).

AMG228 is a IgG1 antibody that binds to GITR and recently completed phase I clinical trial. Of the 30 patients treated, 10 had HNSCC. Of the 29 evaluable patients, none had an objective response. Treatment-emergent toxicity was experienced in 90% of patients; most common being hypophosphatemia, fatigue, anemia, nausea and pyrexia. There was no evidence of altered T cell activity observed despite complete target coverage in both tumor and peripheral blood (32).

The promise of PD-1 inhibitors has been realized for a small proportion of patients with advanced HNSCC. Further work is ongoing, exploring mechanisms of primary and secondary resistance to these agents within HNSCC, aiming to find better predictive biomarkers and methods to increase likelihood for response.

## Combination checkpoint inhibition

Emerging evidence has implicated the upregulation of alternative immune checkpoints in resistance to PD-1/PD-L1 axis interruption (33). The use of combination checkpoint inhibitors (CPI) has been successful in improving response rates and survival in other tumor types, with an increasing number of immune checkpoint inhibitors targeting many co-stimulatory and co-inhibitory interactions transitioning from pre-clinical to clinical evaluation in HNSCC.

CTLA-4 is commonly expressed on the surface of activated T cells, where it binds to B7, preventing interaction with co-stimulatory CD28, leading to negative regulation of T cell proliferation and IL-2 production. Blockade of CTLA-4 correlates with an increase in T-Cell activation and maintenance of high-frequency T-cell receptor clonotypes (34, 35). Dual blockade of PD-L1 and CTLA-4 has been shown to improve response rate and anti-tumor activity when compared to monotherapy alone in metastatic melanoma (36) and the combination is being explored in other solid cancers. In HNSCC, this combination approach is being evaluated in four separate trials (Table 1).

**Table 1:** Ongoing trials evaluating combination PD-L1 and CTLA-4 blockade in HNSCC.

**Trial**	**Phase**	**Cohort**	**Drugs**	**Trial code**
CheckMate 356	I/II	EBV positive nasopharyngeal cancers, HPV positive HNSCC	Ipilimumab and Nivolumab	U111-1166-0687
CheckMate 714	II	Recurrent or metastatic HNSCC	Ipilimumab and Nivolumab vs. Nivolumab and Placebo	NCT02823574
CheckMate 651	III	First line treatment for HNSCC	Ipilumumab and Nivolumab vs. Cetuximab with platinum and flurouracil	NCT02741570
KESTERAL	III	Recurrent or metastatic HNSCC	Durvalumab and Tremelimumab vs. Durvalumab monotherapy	NCT02551159

*Durvalumab, IgG1 antibody to PD-L1; EBV, Epstein-Barr Virus; Ipilimumab, IgG1 antibody to CTLA-4; Tremelimumab, IgG2 antibody to CTLA-4*.

LAG-3 is expressed on the surface of activated CD4+ and CD8+ T-cells and certain subtypes of natural killer and dendritic cells. It is thought to negatively regulate T cell activation and proliferation and is also expressed by Treg cells and required for their optimal suppressive function (37–41). CA224-020 is a phase I/IIa dose escalation and expansion study, exploring BMS-986016 (an anti-LAG-3 antibody) alone and in combination with Nivolumab in advanced solid tumors, including a HNSCC cohort (NCT01968109). Data from the head and neck cohorts are eagerly awaited.

## Checkpoint inhibition in combination with other immune modulators

The immune ecosystem is a complex network of interconnected cells, cytokines and signaling pathways and as such augmenting the antitumor effect of checkpoint inhibitors is not limited to CPI combinations alone. As understanding of this complex biology improves, a number of other agents with the potential to modulate the tumor microenvironment are currently being investigated for the treatment of HNSCC.

SCORES is a phase Ib/II trial of Durvalumab (a PD-L1 inhibitor) in combination with either AZD9150 or AZD5069 in advanced solid tumors including recurrent / metastatic HNSCC (NCT02499328). AZD9150 inhibits STAT3, with pre-clinical evidence of activity in lymphoma and lung cancer (42). Pre-clinical studies have also shown that STAT3 inhibition can increase chemo and radiotherapy sensitization in HNSCC, particularly Nasopharyngeal Cancer (NPC) (43, 44). AZD5069 is a novel selective antagonist of CXC Chemokine receptor 2 (CXCR2), a G protein-coupled receptor for a number of cytokines. It is overexpressed in HNSCC and is implicated in disease proliferation via IL-8 signaling (45, 46). Initial results of patients with HNSCC treated in the AZD9150 and Durvalumab arm were announced at the ESMO Congress 2017. Of 35 patients, 15 had prior PD-L1 treatment and 20 were CPI-naïve. In the CPI- naïve arm, a 25% objective RR was reported (4 confirmed PR and 1 unconfirmed PR) with a 45% DCR at 12 weeks and 30% patients remaining on treatment at 25 weeks. Overall the combination was felt to be tolerable, with G3/4 thrombocytopenia and increase in liver enzymes reported for 3.4% of those dosed, and two treatment related discontinuations (unspecified). These early data are promising and mature results are awaited. In the PD-L1 pretreated arm, 1 complete response and 1 unconfirmed response were reported, with a 20% DCR at 12 weeks (47).

Indoleamine 2,3-dioxygenase 1 (IDO1) is a catabolizing enzyme that induces immune tolerance by suppressing T-cells and has been found to be associated with poor outcome in laryngeal squamous cell carcinoma (48). IDO1 is being heavily investigated as a novel target for immune therapies, with several inhibitors in clinical development. KEYNOTE-037 is a phase I/II study evaluating pembrolizumab given concurrently with epacadostat, an oral inhibitor of IDO1. Patients with recurrent / metastatic HNSCC were eligible for this study if they had received at least one line of platinum based chemotherapy and were CPI-naïve. An interim update of this study was presented at ASCO 2017, when data from 38 patients were presented, 36 of whom were efficacy-evaluable at this early cut off. An ORR of 31% was reported, with a disease control rate of 58%, regardless of the number of previous lines of treatment. The most common treatment related SAEs were fatigue (24%), nausea (11%) and decreased weight (11%). These data suggesting promising anti-tumor activity with good tolerability have led to plans for a phase III study (49).

## Checkpoint inhibition in combination with viral therapy

Oncolytic viruses have been found to reduce tumor burden and prime an anti-tumor immunity in a number of preclinical studies (50, 51) and when used in combination with PD-1 inhibition may overcome CPI resistance by broadening neoantigenome-directed T-cell responses (52). Several oncolytic viruses are currently being evaluated in HNSCC. KEYNOTE-137 is an ongoing phase Ib/III randomized study exploring the combination of Talimogene Laherparepvec (T-VEC) with pembrolizumab in recurrent metastatic HNSCC (NCT02626000). T-VEC is a modified, live, attenuated herpes simplex virus type 1 that is designed to promote an antitumor response through selective replication in tumor cells and production granulocyte macrophage colony-stimulating factor (GM-CSF) to stimulate systemic antitumor immunity. It has already been licensed as a single agent for the treatment of unresectable metastatic melanoma after demonstrating an improved durable response rate (DRR) and mOS relative to GM-CSF (53, 54).

## Checkpoint inhibition in combination with chemoradiotherapy

In addition to interest in combining immune checkpoint inhibitors to other novel agents, there is also a rationale to combine with both chemotherapy and radiotherapy, which are both known to modulate the tumor microenvironment as well as inducing immunogenic tumor cell death. There is particular interest in exploring CPI in combination with chemo-radiation in locally advanced head and neck cancers. Upregulation of PD-L1 by tumor cells following administration of chemo-radiotherapy (CRT) has been demonstrated in a number of preclinical models (55–57) with an improvement in RR, PFS and median time to death being achieved with the addition of adjuvant durvalumab to CRT in locally advanced, unresectable Non-Small-Cell Lung Cancer as demonstrated in the large phase III PACIFIC study (58).

Higher numbers of CD3+ and CD8+ TILs have been shown to positively correlate with clinical outcome to definitive CRT in HNSCC (59). In addition, in a pilot study of 20 patients, CRT was shown to alter the immune landscape in HNSCC, with an increase in the number of CD8+ T effector cells, CD4+ regulatory cells and T cells expressing PD1, TIM3 and LAG3. It is important to note that in this study, most patients were male (90%) with locally advanced human papillomavirus (HPV) associated disease, 80% of which originated from the oropharynx and thus results may not be representative of all HNSCC entities (60).

A safety study demonstrating the tolerability of pembrolizumab in addition to cisplatin-based CRT for locally advanced HNSCC was presented at ASCO 2017. This 27-patient study delivered a fixed dose of pembrolizumab 4–7 days prior to CRT, 3 weekly for the duration of CRT and a further five doses following completion. Patients predominantly had HPV positive oropharyngeal tumors (74%) and all received their planned RT dose, with 85% achieving target cisplatin dosing and 78% completing the planned doses of pembrolizumab. The addition of checkpoint blockade was not felt to significantly increase the toxicity experienced by patients, however three patients did have treatment discontinued due to immune related adverse events (G2 peripheral motor neuropathy, G3 AST elevation and G1 Lhermitte-like syndrome). The study has now progressed into expansion cohorts of both HPV positive and negative tumors to confirm tolerance and gain preliminary evidence of efficacy (61).

Cetuximab is also used with RT for the radical treatment of locally advanced HNSCC. It is an influencer of natural killer cell response and consequently dendritic cell maturation (62, 63) and is thought to increase the expression of inhibitory checkpoints PD-1, TIM-3 and CTLA-4 on TILs (64, 65). There are a number of current trials attempting to determine the benefit of checkpoint inhibition alongside varying combinations of cetuximab and RT in locally advanced HNSCC, with the combination with avelumab as part of REACH (NCT02999087) being of particular interest due to the propensity of both avelumab and cetuximab to activate the antibody-dependent cellular cytotoxicity (ADCC) pathway (63, 66).

## Immune-related adverse events

Treatment with CPIs can have inflammatory side effects which are termed immune related adverse events (irAEs). Although the exact mechanism is unknown, it is likely due to the role that immune checkpoints have in maintaining immune homeostasis and by inhibiting their action, T cells are able to react with self-antigens, with different checkpoint inhibitors having distinct immune toxicity profiles (67). These autoimmune manifestations are more common in patients with pre-existing autoimmune disorders however they may still be safely administered to this population if used with caution and appropriate patient selection (68).

A pooled retrospective analysis of the safety profile of nivolumab in 576 patients with advanced melanoma found the 49% of patients had an irAE; most commonly skin, gastrointestinal, endocrine and hepatic and were classed as grade 3–4 in 4% of patients. The time of onset varied depending on the organ system involved, with skin irAEs manifesting at 5 weeks whereas renal toxicity had had median time of onset of 15 weeks. Approximately 24% required systemic immunosuppressive treatment with the majority of cases resolving (69). In CheckMate 141, the side effect profile of nivolumab in a HNSCC population showed lower rates of both gastrointestinal and hepatic toxicities when compared to the standard of care treatments but did demonstrated an increase in skin toxicity (15.7%), endocrinopathies (7.6%) and pneumonitis (2.1%) (24).

The results of a 114-case series of patients with metastatic HNSCC treated with anti-PD-1 therapy was presented at ASCO 2018, demonstrating that patients who manifested an irAE had improved outcomes compared to those that did not. In total, 59 irAEs were recorded in 49 patients with ORR being higher in irAE positive group (30.6 vs. 12.3% *p* = 0.02) and an improvement in both PFS (6.9 vs. 2.1 months; *p* = 0.0004) and mOS (12.5 vs. 6.8 months; *p* = 0.007) were reported, which remained significant on multivariate analysis (70). A similar observation has been noted in patients with metastatic melanoma and persisted regardless if they required treatment with a systemic immunosuppressant for treatment of their irAE (69).

The development of irAE's can be serious and in some cases fatal, and as such careful consideration must be taken before initiating their use, particularly in the adjuvant or neo-adjuvant setting. As our understanding of the mechanism that drive these systemic manifestations improve, we may develop biomarkers that help identify those who are more likely to develop them which will assist in informed decision making and toxicity monitoring.

## Biomarkers

As improvements in the understanding of the interaction between cancer and the host immunity are complimented by increasing numbers of immunomodulatory drugs, there has been a drive to develop potential biomarkers to select patients most likely to benefit from treatment and assist in monitoring response.

Both the nivolumab and pembrolizumab studies outlined above explored the impact of HPV on outcome. In Checkmate-141, OS appeared to be longer with nivolumab regardless of p16 status, however the increase was more pronounced in patients with p16 positive tumors (mOS of 9.1 months with nivolumab vs. 4.4 months with standard therapy) than in p16 negative tumors (mOS 7.5 vs. 5.8 months respectively) (24). KEYNOTE-012 also reported better outcomes in the patients with HPV-positive tumors relative to HPV negative ones (RR 32% vs. 14%, 6 month PFS 37% vs. 20% and 6-month OS 70% vs. 56% (28).

These trials also examined tumor cell PD-L1 expression as a potential biomarker, with data suggesting increased benefit in PD-L1 positive disease. In the overall survival analysis of Checkmate-141, patients with PD-L1 expression >1% treated with nivolumab had a hazard ratio for death of 0.55 (95% CI 0.36–0.83) when compared to standard therapy. Where PD-L1 expression was < 1%, this HR was 0.89 (95% CI 0.54–1.45) (24). Similarly, KEYNOTE-012 reported tumor PD-L1 expression using immunohistochemistry, with positivity being defined as >1%. Patients with PD-L1 expression >1% who received pembrolizumab had improved RR of 22 vs. 4% in those with PD-L1,1%, with median OS 303 vs. 151 days respectively (28). KEYNOTE-040 described an OS HR of 0.54 (95% CI 0.35–0.82) with pembrolizumab in patients with tumors with PD-L1 expression >50% but also evaluated PD-L1 expression on both tumor and associated immune cells (CPS score) describing OS HR of 0.75 for PD-L1 CPS >1% with pembrolizumab compared to control patients.

Of the 61 PD-L1 positive HNSCC in KEYNOTE-012, 43 had RNA expression profiling and survival data were evaluated with multi-gene expression signatures that had previously been derived in melanoma patients. Of these signatures, the 6-gene INF-γ was the top-performing, with significant associations to OR (*p* = 0.005) and PFS (<0.001). On evaluation of the individual signature genes, INF-γ inducible MHC-II expression was felt to be the biological link. Using an optimal cutoff for INF-γ, positive predictive value for response was 40% with a negative predictive value of 95%; AUC = 0.8 (95%CI 0.61–0.95) within this patients population, which may assist in identifying clinical benefit from anti-PD-1 therapy in patients who are PD-L1 positive (71).

The somatic mutational load (ML) and INF-γ gene expression profile were found to be independently predictive of response to pembrolizumab in the 73 patients within KEYNOTE-012 who had HPV and Epstein-Barr Virus (EBV) negative HNSCC, with ML and INF-γ gene expression profile being significantly associated with OR (*p* = 0.064 and *p* = 0.001; AUROC 0.82 and 0.74 respectively). The INF-γ gene expression profile also remained a significant predictor in HPV and EBV positive patients showing promise as a predictor of response regardless of viral status (72).

Combinations of these biomarkers may give additive value to patient selection for CPI therapy. Other potential biomarkers that have shown promise include epigenetic modification of genes associated with homologous recombination, such as RAD51 and XRCC3, which are thought to alter checkpoint expression (73) and the identification of different HNSCC subtypes, each with a distinct tumor microenvironment (74) but how these influence survival or response to immunotherapy has yet to be addressed.

## Conclusion

Immunotherapy looks set to revolutionize the treatment of HNSCC, with the approval of nivolumab and pembrolizumab (FDA) already offering new therapeutic options in recurrent / metastatic disease. As our knowledge of the biological processes driving HNSCC improves, along with greater understanding of the important features of the tumor microenvironment, so too does the rationale for combination strategies and the parallel development of predictive biomarkers. These approaches should support an era where a personalized approach to immunotherapy treatment translates into improved outcomes for patients with this disease.

## Author contributions

Both M-JD and MF were involved in selecting the content and outline and the writing of this article.

### Conflict of interest statement

The authors declare that the research was conducted in the absence of any commercial or financial relationships that could be construed as a potential conflict of interest.
